# Outcome of transcatheter atrial septal defect closure in a nationwide cohort

**DOI:** 10.1080/07853890.2023.2178669

**Published:** 2023-02-14

**Authors:** V. Muroke, M. Jalanko, J. Haukka, J. Hartikainen, A. Tahvanainen, H. Ukkonen, K. Ylitalo, J. Pihkala, J. Sinisalo

**Affiliations:** a Department of Cardiology, Helsinki University Hospital, Helsinki, Finland; b Department of Public Health, University of Helsinki, Helsinki, Finland; c Department of Cardiology, Kuopio University Hospital, Kuopio, Finland; d Department of Cardiology, Tampere University Hospital, Tampere, Finland; e Heart Centre, Turku University Hospital, Turku, Finland; f Department of Cardiology, Oulu University Hospital, Oulu, Finland; g Department of Cardiology, New Children’s Hospital, Helsinki University Hospital, University of Helsinki, Helsinki, Finland

**Keywords:** Atrial septal defect, congenital heart disease, atrial fibrillation, stroke, migraine, complication, transcatheter closure

## Abstract

**Background:**

Transcatheter (TC) atrial septal defect (ASD) closure has been the mainstay of therapy for secundum-type ASDs for over 20 years.

**Aims:**

This nationwide cohort evaluated the long-term outcome of transcatheter-closed ASDs.

**Methods:**

The study enrolled every transcatheter ASD closure performed in Finland from 1999 to 2019. Five age, sex, and municipality-matched controls per ASD patient were gathered from the general population. The median follow-up period was 5.9 years (range 0–20.8). We used the hospital discharge register to gather all hospital visits and diagnoses. Closure complications and echocardiographic changes were collected from the electronic health records.

**Results:**

Transcatheter ASD closure was performed in 1000 patients (68.5% females) during the study period. The median (range) age at the time of the procedure was 37.9 (1.8–87.5) years. ASD patients had an increased risk for new-onset atrial fibrillation (RR 2.45, 95% CI: 1.84–3.25), migraine (RR 3.61, 95% CI: 2.54–5.14), ischemic heart disease (RR 1.73, 95% CI: 1.23–2.45), ventricular fibrillation/tachycardia (RR 3.54 (95% CI: 1.48–8.43) and AV conduction disorder (RR 3.60, 95% CI: 1.94–6.70) compared to the control cohort. Stroke risk was not increased (RR 1.36, 95% CI: 0.91–2.03). Adverse events occurred in 6.3% (*n* = 63) of the patients, including four erosions and ten device embolizations.

**Conclusion:**

After TC closure of ASD, patients had a higher risk of new-onset atrial fibrillation and migraine ***than controls without ASD***. As novel findings, we found an increased risk for ischemic heart disease, AV conduction disorders, and ventricular fibrillation/tachycardia.

**Key messages**
- Even though patients have an excellent overall prognosis after percutaneous ASD closure, the increased incidence of major comorbidities like atrial fibrillation and heart failure prompts more thorough lifelong follow-up.
- This study’s novel findings revealed the increased risk for ischemic heart disease, AV conduction disorders, or ventricular tachycardia/fibrillation during the follow-up.
- Major complications after the closure are rare; erosion is seen in 0.4% of the patients and embolization in 1.0% of the patients.

## Introduction

Atrial septal defect (ASD) is the second most common congenital heart defect, with a birth prevalence of 2.9/1000 [1]. Secundum-type ASD causing right ventricular volume overload is usually treated with transcatheter (TC) device closure.

Patients with device ASD closure have an excellent prognosis, with low short- and long-term mortality of (0.01% and 0.1%, respectively) [2]. The rate of major complications in transcatheter closure is estimated to be 1.6% [2] and the pooled rate of minor and major complications is 7% [3].

ASD patients have an increased risk for atrial fibrillation, stroke, and migraine. The rate of new-onset atrial fibrillation remain elevated even after ASD closure. The risk of stroke is also increased in both patients with and without closure [4]. Migraine can worsen in the first months after the closure [5]. However, there is no clear consensus on whether TC-closed ASD patients have an increased risk of stroke or migraine in the long run [4,6].

Catheter-based ASD closure has been a mainstay therapy for secundum-type ASDs for over 20 years, and up to 80–90% of defects are deemed eligible for catheter closure [7,8]. We are starting to get long-term results of the procedure’s outcome. Numerous studies have reported excellent prognosis and low complication rates in patients with TC-closed defects [2,6,9–11]. However, there is limited data about the disease burden in the long run after closure.

In this study, we report the long-term outcome of all Finnish ASD patients who underwent device closure by the end of 2019.

## Methods

### Study population

We retrospectively gathered data on all TC ASD closures performed in Finland from 1999 to 2019. All procedures were performed at five different tertiary centers. Patients were identified from the hospital procedure registers. Only cases that were stated as an atrial septal defect by echocardiographer were included. Defects that resembled more patent foramen ovale were not included in the analysis. Detailed information about the baseline comorbidities, closure-related information, complications, and one-year follow-up visit were gathered from the electronic health records (EHR).

Each ASD patient was matched with five individuals from the register of the digital and population data services agency were matched for each ASD patient based on birth year, residency at the date of closure, and sex. Dates of all hospital visits and ICD-10 diagnosis codes were gathered from the Finnish hospital discharge register. Mortality data, including the date of death and causes of death by ICD-10 codes, were obtained from Statistic Finland.

### Complications and disease burden

Incidences of new-onset atrial fibrillation (AF), stroke, heart failure, and migraine were gathered from the hospital discharge registry. Patients with a history of AF, stroke, heart failure, or migraine prior to ASD closure were excluded from the analysis.

Complications that could be directly linked to the closure procedure or the closure device and leading to death, disability, morbidity, medical intervention, or prolonged hospital stay were gathered from the electronic health records and Statistics Finland.

### Echocardiography, ECG, and shunt size measurement

Echocardiography was performed, and ECGs were obtained from all patients before the ASD closure and during the 1-year follow-up visit. Valvular regurgitations were graded as mild, moderate, or severe. Left (LV) and right ventricle (RV) systolic function, right heart size, estimated pulmonary pressure, and the incidence of valvular regurgitations before and after the closure were recorded.

The ASD size was measured using TEE. Values are reported as maximum diameter. Right heart size was measured using different methods depending on the institution. Combined right atrium (RA) + RV measurement during end diastole was used in 218 patients, RV size was measured from parasternal long axis view in 236 patients, and basal RV end-diastolic diameter in 597 patients.

Most commonly, shunt size (Qp/Qs) was measured by non-invasive dye dilution (40.9%, *n* = 227) [12], followed by the radionuclide method (38.7%, *n* = 215). The rest of the shunts were measured using cardiac magnetic resonance (CMR) or invasive oximetry. Few pediatric (*n* = 4) patients had shunt size estimated using TTE.

### Statistical analysis

The results are expressed as mean (standard deviation) or median (interquartile range). Counts and percentages were used to summarize categorical variables. Cumulative events of different events were plotted using Kaplan–Meier estimates. Poisson regression with 95% confidence intervals was used to calculate incidence risk ratios (RRs) based on first-event incidence rates. Risk ratio adjustments were performed on age, year, sex, and Charlson comorbidity index at the time of the ASD closure. The index did not include conditions that could have been caused by ASD, such as cerebrovascular disease and hemiparesis. All the reported risk ratios are adjusted. Numerical values of crude risk ratios are shown in Supplemental Table 2.

The Chi-square test or Mann-Whitney *U*-test was used to compare baseline characteristics. A paired *t*-test was used to compare echocardiographic changes during follow-up. All analyzes were performed using R software, version 4.2.1.

### Ethics

The Helsinki University Hospitals Ethics Committee accepted this study on 11.7.2019 (number HUS/1820/2019), which was carried out in accordance with the Helsinki Declaration. For register-based research, no patient permission was required.

## Results

### Baseline characteristics

In total, 1000 patients with 1019 TC ASD closure procedures between 1999 and 2019 were included. The median age at the time of closure was 37.9 (IQR 11.1–57.3, range 1.8–87.5), and 32,3% (*n* = 323) of patients were under 18 years old (Table 1). The median follow-up was 5.9 years (IQR 2.3–10.5, range 0–20.8), resulting in a total follow-up time of 8248 person-years.

**Table 1.** Descriptive characteristics: Values are presented as mean (SD), median (IQR), or percentage (number of patients). Data is based on electronic health records.

	ASD-patients	Missing
	*N =* 1000	
Female (%)	68.5% (685)	0
Median age at the time of the procedure	37.9 (11.1–57.3)	0
Median BMI	23.2 (17.7–27.4)	329
Another congenital heart defect:	7.1% (71)	3
AF:		6
Chronic	10.9% (108)	
Paroxysmal	6.4% (64)	
SVT	3.0% (30)	6
History of VT/VF	0.5% (4)	0
HFrEF	1.8% (18)	3
Migraine	7.8% (77)	18
Stroke	12.4% (124)	4
TIA	3.1% (31)	4
NYHA class		
1	78.0% (750)	38
2	17.4% (167)	
3	4.6% (44)	
4	0.1% (1)	
Multi fenestrated ASD	13.5% (135)	2
Floppy septum	20.2% (90)	555
Mean Qp/Qs ratio	2.0 (0.59)	445
Median native ASD size on TEE (mm)	11.6 (8.5–15)	122
Median balloon size (mm)	15.5 (12–20)	64
Brand name of the device		7
Amplatzer ASD	84.8% (842)	
Amplatzer Cribriform	1.8% (18)	
Amplatzer PFO	3.3% (33)	
Figulla	4.7% (47)	
HELEX	4.4% (44)	
STARflex	0.9% (9)	
Amplatzer ASD size:		159
<20 mm	66.0% (555)	
20–30 mm	31.6% (266)	
≥30 mm	2.4% (20)	
Post op antithrombotic		16
Acetylsalicylic acid	74.7% (735)	
DOAC or warfarin	14.2% (140)	
Dual antiplatelet therapy	5.5% (54)	
Anticoagulant + antiplatelet	2.8% (28)	

AF: Atrial fibrillation; ASD: atrial septal defect; BMI: body mass index; DOAC: Direct oral anticoagulant; HFrEF: Heart failure with reduced ejection fraction; NYHA: New York Heart Association; PFO: Patent foramen ovale; SVT: Supraventricular tachycardia; TIA: Transient ischemic attack; VF: Ventricular fibrillation; VT: Ventricular tachycardia.

Data about septum mobility was available for 445 patients, and 20.2% of those (*n* = 90) had a floppy septum. Floppy septa were more often multi-fenestrated (18.9% vs. 10.1%, OR 2.1, 95% CI:1.1–4.0). Patients with a floppy septum had a more often history of stroke (37.8% vs. 14.5%, OR 3.6, 95% CI:2.1–6.0) and migraine (19.1% vs. 10.3%, OR 2.1, 95% CI:1.1–3.8) before the ASD closure.

NYHA class was normal (NYHA 1) in 96.9% of patients under 18. Exertional limitation increased with age, with 43.7% of patients over 50 reported NYHA class 2–4. Adult patients with NYHA class 3–4 had larger defects than NYHA 1-2 patients (19.1 mm vs. 16.9 mm, respectively, *p* = 0.03).

All patients had antithrombotic medication after the closure. 74.7% were on acetylsalicylic acid 100 mg o.d. (in children, approximately 5 mg/kg o.d.). The duration of the medication was six months in 75.1% of the study population, 22.1% were prescribed lifelong antithrombotic therapy, and the rest had a duration of 1, 3, or 12 months.

### Morbidity and hospitalizations

**Figure 1.**
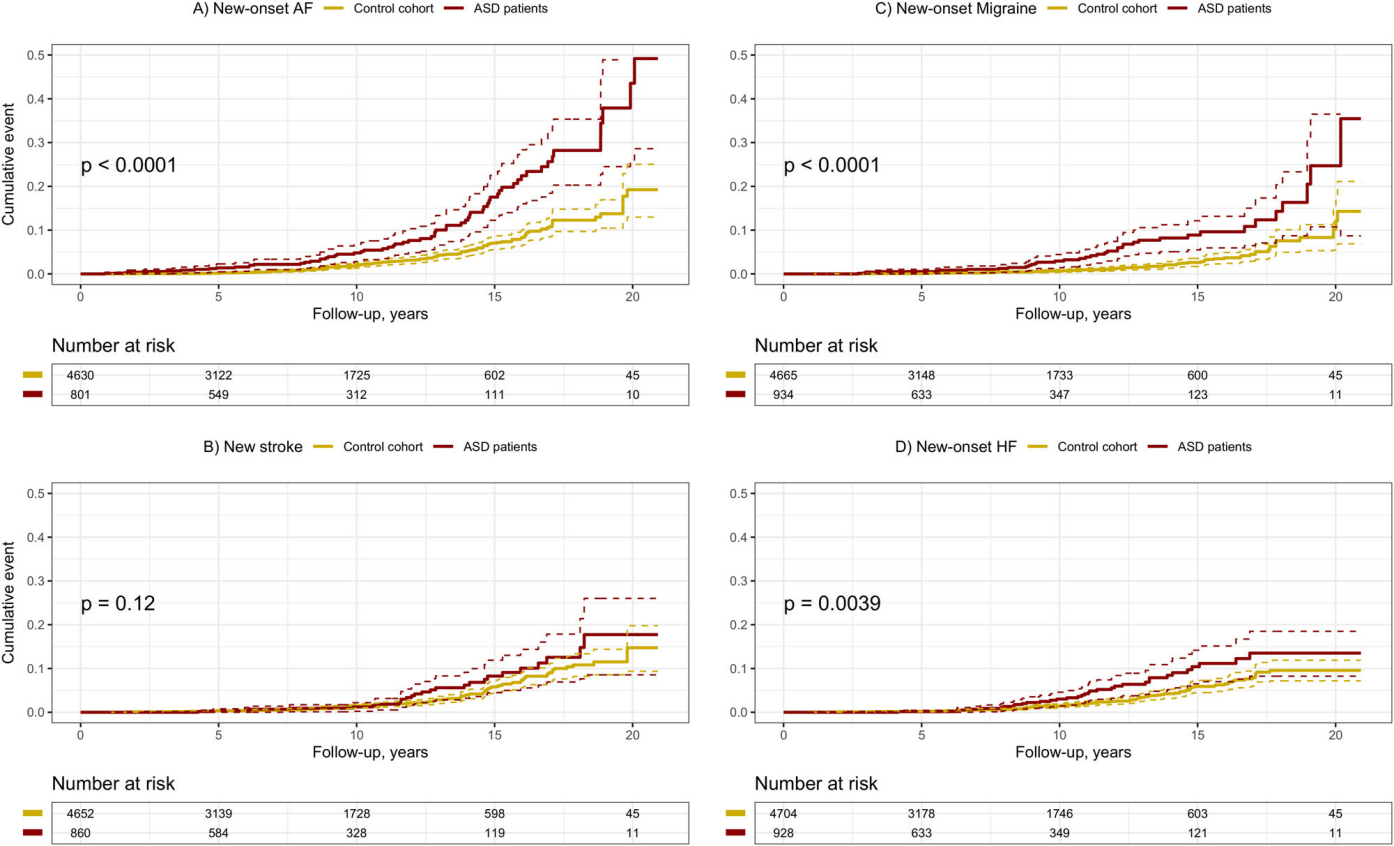
Cumulative incidence of (A) New-onset AF, (B) New ischemic stroke, (C) New-onset migraine and (D) New-onset heart failure after the closure during up to 20 years of follow-up. Data is based on the diagnoses from the hospital discharge registry.

The incidence of new-onset atrial fibrillation/flutter (AF) was 9.2% (*n* = 74) during the follow-up, RR 2.45 (95% CI: 1.84–3.25) compared to the control cohort. The risk ratio for new-onset AF was 13.85 (95% CI: 6.88–27.88) in 18- to 50-year-old patients and 1.54 (95% CI: 1.09–2.17) in over 50-year-old patients (Supplemental appendix, Figure 1). New-onset heart failure was diagnosed in 3.8% (*n* = 36) of the ASD patients and there was no difference in the risk ratio after the adjustments (RR 1.43, 95% CI: 0.97–2.10) (Figure 1).

**Figure 2.**
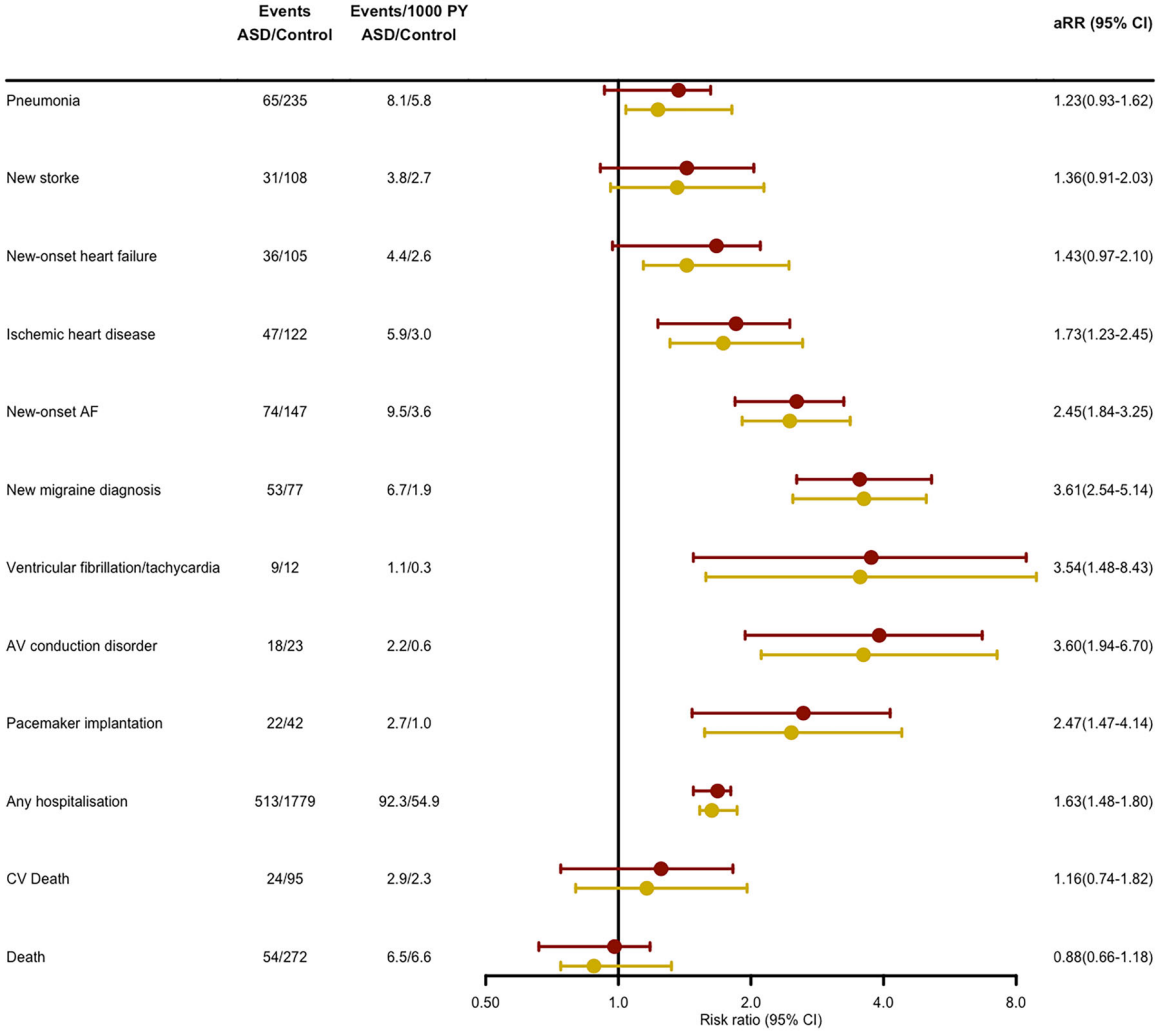
Long-term outcomes and incidence risk ratios for events during the follow-up after percutaneous ASD closure. Red lines represent adjusted risk ratios and yellow lines crude risk ratios. AF: atrial fibrillation, ASD: Atrial septal defect; AV: atrioventricular; CV: Cardiovascular; PY: Person year; aRR: Adjusted risk ratio.

In total, 31 ASD patients experienced a new-onset stroke during the follow-up. There was no difference in the incidence of new-onset stroke compared to the control population (RR 1.36, 95% CI: 0.91–2.03) (Figure 2). The risk ratios for stroke were 2.45 (95% CI: 1.05–5.70) and 1.23 (95% CI: 0.78–1.95) in patients aged 18–50 and over 50-year-old, respectively (Supplemental appendix Figure 2). Nine of the 31 stroke patients had atrial fibrillation diagnosed before the ASD closure and six patients developed new-onset atrial fibrillation during the follow-up. New-onset migraine was found in 53 ASD patients during the follow-up, and the risk ratio for migraine was 3.61 (95% CI: 2.54–5.14) compared to the control population.

ASD size was similar in patients with or without new-onset atrial fibrillation (16.1 mm vs. 16.4 mm, *p* = .79), migraine (16.0 mm vs. 17.7 mm, *p* = .053) and VT/VF (18.8 mm vs. 16.3 mm, *p* = .42) during the follow-up. Patients with new-onset stroke or new-onset heart failure had larger ASDs by stop-flow balloon measurement; 18.4 mm vs 15.5 mm (*p* = .0001) and 18.5 mm vs. 15.9 mm (*p* = .045) respectively.

We found no significant difference in the baseline shunt in patients with or without new-onset atrial fibrillation (1.95 vs. 2.06, *p* = .12), new-onset stroke (2.03 vs. 2.10, *p* = .28), new-onset heart failure (1.99 vs. 1.95, *p* = .99) or new-onset migraine (2.01 vs. 2.05, *p* = .5).

Compared to the reference population, ASD patients had an increased risk for ischemic heart disease (RR 1.73, 95% CI: 1.23–2.45), ventricular rhythm disturbances (RR 3.54, 95% CI: 1.48–8.43), and atrioventricular (AV) conduction disorder (RR 3.60, 95% CI: 1.94–6.70). Increased risk for AV conduction disorder was also reflected in the increased incidence of pacemaker implantations (RR 2.47, 95% CI: 1.47–4.14).

Compared to the reference population the incidence of hospitalizations was higher in the ASD group (RR 1.63, 95% CI: 1.48–1.80) (Figure 2). When the 30 d following the closure were excluded, the hospitalization risk ratio was 1.49 (95% CI: 1.35–1.65).

**Table 2.** Complications during the procedure, hospital stay, and after the discharge.

	During procedure	During hospital stay	After discharge
Major bleed	1 (0.1%)		4 (0.4%)
Device embolization	2 (0.2%)	6 (0.6%)	2 (0.2%)
Device erosion		1 (0.1%)	3 (0.3%)
Catheter thrombus	11 (1.1%)		
Ruptured septum	1 (0.1%)		
Coronary occlusion	1 (0.1%)		
Access site complication	1 (0.1%)	10 (1.0%)	9 (0.9 %)
Intubation related complication	5 (0.5%)		
Pericardial effusion		3 (0.3%)	
Post op. fever		4 (0.4%)	
Allergic reaction	1 (0.1%)	1 (0.1%)	1 (0.1 %)

### Complications

Adverse events occurred in 6.3% (*n* = 63) of the patients. Altogether, access site complication was the most prevalent complication occurring in 2.0% (*n* = 20) of patients. A procedure-related complication was recorded in 2.3% of the patients, most commonly catheter thrombus (1.1%). Complication during the hospital stay was observed in 2.5% of the patients, and 1.9% had complications after discharge. All erosions and embolizations occurred in adult patients (Table 2).

**Table 3.** ECG and echocardiographic changes before and in a mean follow-up of 10 months after percutaneous ASD closure.

	Baseline	Post	Mean of the difference (95% CI)	*p*-value
AF on ECG:	6.21% (55)	7.59% (34)	−1.4% (−4.3% to 1.5%)	.267
RBBB or pRBBB on ECG	49.2% (384)	18.9% (82)	30.3% (25.2% to 35.4%)	<.001
LV EF	64.2 (8.85)	65.2 (7.25)	−0.96 (−0.21 to −1.71)	.012
TAPSE	25.9 (6.02)	23.7 (5.12)	2.38 (0.71 to 4.05)	.0058
ASD flow on TTE	85.4% (677)	8.87% (59)	76.5% (73.2% to 79.8%)	<.001
Aortic regurgitation				
Mild	6.22% (55)	6.75% (41)	−0.5% (−3.1% to 2.0%)	.68
Moderate	0.57% (5)	0.82% (5)	−0.3% (−1.1% to 0.6%)	.55
Severe	0.0% (0)	0.0% (0)	NA	NA
Mitral regurgitation				
Mild	18.6% (165)	25.3% (154)	−6.7% (−11% to −2.4%)	.002
Moderate	2.47% (22)	2.30% (14)	0.2% (−1.4% to 1.7%)	.83
Severe	0.11% (1)	0.00% (0)	0.1% (−0.1% to 0.3%)	.41
Tricuspid regurgitation				
Mild	52.1% (440)	58.7% (341)	−7.5% (−12.7% to −2.3%)	.005
Moderate	8.10% (70)	4.79% (28)	3.3% (0.8% to 5.8%)	.014
Severe	1.16% (10)	0.68% (4)	0.5% (−0.5% to 1.4%)	.37
TI gradient	30.3 (11.5)	25.2 (9.55)	5.59 (4.22 to 6.96)	<.001
Enlarged right side	83.9% (745)	24.4% (138)	59.5% (55.2% to 63.8%)	<.001
RV size (RV + RA area)	45.5 (13.5)	34.6 (11.4)	13.16 (10.07 to 16.24)	<.001

AF: Atrial fibrillation; ASD: atrial septal defect; LV EF: Left ventricular ejection fraction; NA: Not applicable; pRBBB: Partial right bundle branch block; RA: Right atrium; RBBB: Right bundle branch block; RV: Right ventricle; TAPSE: Tricuspid annular plane systolic excursion; TI: Tricuspid valve insufficiency; TIA: Transient ischemic attack; VF: Ventricular fibrillation; VT: Ventricular tachycardia.

Values are presented as mean (SD) for continuous variables or percentage (number of patients) for categorical variables.

The 30 d mortality after the closure was zero percent, and no deaths were associated with the closure procedure. In the total study sample, prolonged (more than one night) hospital stay was noted in 2.6% (*n* = 26) of the patients. Hospital stay was more often prolonged in adults compared to pediatric patients (3.4% vs. 0.9%, *p* = .036).

Aortic rim data was available for 437 patients. The aortic rim was absent in 7.1% (*n* = 31) patients and deficient in 31.4% (*n* = 137) patients. They did not have increased complication rates during the procedure (*p* = .65), during the hospital stay (*p* = .20) or after the discharge (*p* = .26). Patients with absent aortic rim had no erosions or embolizations.

### Echocardiography

Repeat echocardiography was performed during the follow-up visit (mean follow-up of 10 months). The right heart size decreased during the follow-up. The dilated right heart was present in 83.9% of the patients before the closure and in 24.4% after the closure. There was a significant (*p* < .001) difference in RV normalization between age groups (Table 3). At the control visit, RV remained enlarged in 11.2%, 17.7%, and 43.1% of the patients in the under-18-year-old, 18–50-year-old, and over-50-year-old groups, respectively (Supplemental appendix, Table 1). Residual shunting at the atrial level was seen in 8.9% of patients in the control visit. Only one patient required re-operation because of the residual shunt.

## Discussion

In this comprehensive study, we were able to get versatile long-term follow-up information by combining detailed EHR data with the Finnish hospital discharge registers data.

### Morbidity

ASD patients were at increased risk of developing new-onset atrial fibrillation and migraine after the closure. As novel findings, we found a higher risk for ischemic heart disease, AV conduction disorders, and ventricular fibrillation/tachycardia.

In the previous study, we found that ASD patients had an increased risk for ischemic heart disease mortality [13]. In line with that finding, we demonstrated an increased risk of new-onset ischemic heart disease during the follow-up in the current (RR 1.73). The higher frequency of ischemic heart disease could be due to associated ischemic cardiomyopathy that could increase the left-to-right shunt and reveal the clinical picture. Giannankoulas et al. found that severe coronary artery disease frequency in the adult congenital heart disease (ACHD) group was comparable to the general population [14]. However, when comparing different congenital heart diseases, ASD is the most prevalent congenital heart defect in ACHD patients with coronary artery disease [15,16].

The proximity of the AV node to the ASD rims puts it at risk of injury after the device implantation. Indeed, we found that the risk for AV conduction disorder was 3.91 times higher in the ASD population compared to the controls. AV block has been reported to develop in 0.4–4.5% of patients undergoing ASD closure, but to our knowledge, the higher relative risk compared to controls has not been reported previously [2,17]. The higher incidence of AV conduction disorders may have manifested as an elevated risk for pacemaker implantation (RR 2.47). The higher risk for pacemaker implantation was not found in a similar-sized study by Abrahamyan et al. [6]

Ventricular rhythm disturbances were more common in the ASD cohort (RR 3.54). It is known that ASD patients have more nonsustained ventricular tachycardias (NSVT) in the Holter recordings (8% vs. 0.7% in the general population) [18]. However, to our knowledge, there are no previous reports of the increased rate of VF/VT after percutaneous ASD closure. Right ventricular volume overload and strain is the most probable explanation for this finding.

In our study, 9.2% of patients developed new-onset AF after the ASD closure, with a risk ratio of 2.45 compared to the matched reference population. A Danish cohort study reported a higher hazard for new-onset atrial fibrillation (HR 8.2) after the closure during a mean follow-up of 9.6 years [4]. Abrahamyan et al. showed new-onset atrial fibrillation incidence of 14.9% after the closure in 10.6 years of follow-up [6] and in a study by Doung et al., the incidence of new-onset AF was 6% in patients over 40 years old during a shorter follow-up of 3.6 years [19].

The lower rate of atrial fibrillation in our study is most probably explained by the inclusion of both adult and pediatric patients (32.3% of the whole cohort) since only two pediatric patients had AF during the follow-up.

A meta-analysis of 31 studies by Himelfarb et al. concluded that the incidence of new-onset atrial fibrillation was relatively low after transcatheter ASD closure [20]. These studies had an average follow-up duration of 0.5–9 years, and the overall incidence of new-onset AF was 1.19 (95% CI: 0.75–1.89) per 100 person-years. Our results align with this meta-analysis with the incidence of new-onset AF of 0.95 per 100 person-years in the whole population and 1.5 per 100 person-years in adult patients.

Even though atrial fibrillation is a well-known risk factor for stroke, we did not see an increased risk for ischemic stroke. A similar finding was found by Abrahamyan et al. [6]. In the study by Nyboe et al. the risk of stroke was increased after the closure with an HR of 2.0 [4]. In their study, this risk was explained by the increased AF incidence. In our study, 12% of the patients had experienced a stroke before the closure. Similar finding were also found by Dolgner et al. In their research, 1% of patients had a stroke during 12 months of follow-up, and all the new-onset stroke patients had atrial fibrillation [21].

The association between ASD and migraine has been reported previously [22,23]. It is well known that already existing migraine can worsen in the first month following the ASD closure [5]. In our study, we showed that the risk for new-onset migraine was markedly increased (RR 3.61) even after the shunt was closed, and the risk seemed to remain elevated in the long-term follow-up. In a Danish register-based study, the hazard ratio for new migraine was even higher (HR 5.5) after the ASD closure [23]. The rate of new-onset migraine was 8.2% in our study, which is consistent with previous studies (0–24%) [24].

The mechanism for the increased risk of post-procedure migraine remains unknown. It is hypothesized that the device can cause a local inflammatory reaction and result in platelet adhesions, which could then embolize to the brain, causing microinfarcts and migraine headaches. Another explanation could be the release of inflammatory mediators into the left atrium, which would travel to the cerebral circulation and cause migraine headaches [25]. We also know that bigger ASD size and lower plasma calcinogen-related peptide (CGRP) levels prior to closure are associated with new-onset migraine [26].

### Complications

In total, the rate of complications was low, and similar complication rates have been reported previously. Device erosion developed in 0.4% of the cases, and device embolization in 1.0% of patients. Both findings are in line with previous studies [2,7,9,27]. The incidence of embolization was 0.62%, and the incidence of erosion was 0.28% in the US Food and Drug Administration registry’s analysis [10].

### Echocardiography

We found a decrease in moderate to severe tricuspid regurgitations. This finding was somewhat expected since the right heart size decreases after the closure, which decreases functional tricuspid regurgitation. The severity of tricuspid regurgitation decreased from moderate-severe to mild in 40.8% of the patients with moderate to severe TR before the closure. In a study with a longer follow-up of 30 months, the severity of TR decreased from moderate-severe to mild in 70% of the patients [28].

We saw an increase in the incidence of mitral regurgitation after the closure, which was caused solely due to a higher incidence of mild mitral regurgitation. Thus, this finding likely has little to no clinical significance. It has been speculated that the worsening of the mitral regurgitation is caused by decreased RV size and concomitant LV size enlargement and mitral annulus modification [29,30]. A review study by Jalal et al. concluded that mitral regurgitation (MR) may occur or worsen in 10–37% of patients after percutaneous ASD closure. They also found that this finding was usually trivial to moderate [31]. There are also reports of improvement of pre-existing mitral regurgitation after the procedure [32].

As expected, right heart size normalized after ASD closure in most cases. However, if the defect was closed after the age of 50, the right heart size remained enlarged in half of the patients, demonstrating the need for early intervention. Similar results have been reported in patients with surgical ASD closure [33]. A residual shunt was seen rather commonly (8.9%). However, the shunts were very small and, in most cases, known beforehand (e.g. a multifenestrated defect that could not be closed entirely). Only one patient needed to be operated on for a residual shunt.

### Limitations

The study’s retrospective nature limited a more thorough evaluation of the incidence of different outcomes during the follow-up. Due to the nature of the registry, we could only trust the first occurrence of the diagnosis and thus could not study the incidence of AF burden or recurrence of strokes.

We do not have information from diagnoses made in primary care or by general practitioners because the hospital discharge registry only contains data from hospital visits. As a result, some of the outcomes may be missed. This accounts mainly migraine since strokes, atrial fibrillations, ischemic heart disease, and AV-conduction disorders are diagnosed and treated in a hospital setting.

Follow-up can be considered nearly complete regarding the hospital visits, mortality, and morbidity since only patients that have moved abroad were lost to follow-up. However, some echocardiographic parameters were unavailable at a one-year follow-up visit.

## Conclusion

TC ASD closure is a safe procedure with low complication rates. However, patients undergoing TC ASD closure have an increased risk for new-onset atrial fibrillation, ischemic heart disease, AV conduction disorder, ventricular fibrillation/tachycardia, and migraine during the long-term follow-up.

## Supplementary Material

Supplemental Material

## Data Availability

The data supporting this study’s findings are available from Statistics Finland and the FHDR. Still, restrictions apply to data availability, which was used under license for the current research and is not publicly available. Data are, however, available from the authors upon reasonable request and with permission from Statistics Finland and the FHDR.
