# Supplementary material for: Outcome of transcatheter atrial septal defect closure in a nationwide cohort
Source: Ann Med. 2023 Feb 14;55(1):615–23. doi: 10.1080/07853890.2023.2178669 (PMC9930864; doi:10.1080/07853890.2023.2178669)

Supplemental appendix

Supplemental table 1 : ECG and echocardiographic changes before and after the closure in different age groups

|  | <18  (*n=323)* | 18-50 (n=316) | >=50  (n= 361) | p.overall |
| --- | --- | --- | --- | --- |
| AF on ECG: |  |  |  |  |
| Baseline | 0 (0.00%) | 1 (0.37%) | 54 (18.1%) | <0.001 |
| Post | 0 (0.00%) | 0 (0.00%) | 34 (27.0%) | <0.001 |
| RBBB or pRBBB on ECG: |  |  |  |  |
| Baseline | 154 (66.7%) | 97 (36.3%) | 133 (47.2%) | <0.001 |
| Post | 35 (17.8%) | 16 (13.4%) | 31 (26.5%) | <0.001 |
| EF |  |  |  |  |
| Baseline | 66.7 (7.26) | 64.1 (7.06) | 62.1 (10.8) | <0.001 |
| Post | 65.9 (6.33) | 64.8 (6.87) | 65.0 (8.23) | 0.225 |
| TAPSE |  |  |  |  |
| Baseline | 25.1 (6.07) | 26.1 (6.33) | 25.9 (5.72) | 0.652 |
| Post | 22.8 (4.83) | 24.7 (5.53) | 23.2 (4.69) | 0.113 |
| ASD flow on TTE |  |  |  |  |
| Baseline | 315 (99.7%) | 180 (78.3%) | 182 (73.7%) | <0.001 |
| Post | 11 (5.45%) | 20 (9.22%) | 28 (11.4%) | 0.087 |
| Aortic regurgitation |  |  |  |  |
| Baseline mild | 2 (0.63%) | 11 (4.15%) | 42 (14.0%) | <0.001 |
| Baseline moderate | 0 (0.00%) | 1 (0.38%) | 4 (1.33%) |  |
| Post mild | 3 (1.45%) | 8 (4.40%) | 30 (13.8%) | <0.001 |
| Post moderate | 0 (0.00%) | 0 (0.00%) | 5 (2.29%) |  |
| Mitral regurgitation |  |  |  |  |
| Baseline mild | 8 (2.50%) | 55 (20.5%) | 102 (33.9%) | <0.001 |
| Baseline moderate to severe | 0 (0.00%) | 4 (1.49%) | 19 (6.3%) |  |
| Post mild | 21 (10.1%) | 40 (21.7%) | 93 (42.7%) | <0.001 |
| Post moderate | 0 (0.00%) | 3 (1.63%) | 11 (5.05%) |  |
| Tricuspid regurgitation |  |  |  | . |
| Baseline mild | 97 (30.1%) | 169 (67.1%) | 184 (62.8%) | <0.001 |
| Baseline moderate to severe | 4 (1.25%) | 12 (4.76%) | 64 (21.8%) |  |
| Post mild | 92 (45.3%) | 107 (60.5%) | 144 (69.9%) | <0.001 |
| Post moderate to severe | 2 (0.99%) | 5 (2.86%) | 25 (12.1%) |  |
| TI gradient |  |  |  |  |
| Baseline | 25.7 (9.18) | 26.7 (9.77) | 33.8 (12.0) | <0.001 |
| Post | 23.1 (8.28) | 21.5 (7.48) | 28.0 (10.0) | <0.001 |
| Enlarged right side: |  |  |  |  |
| Baseline | 279 (92.7%) | 204 (74.2%) | 262 (84.0%) | <0.001 |
| Post | 21 (11.2%) | 32 (17.7%) | 85 (43.1%) | <0.001 |
| RV + RA area |  |  |  |  |
| Baseline | 33.9 (6.19) | 42.3 (12.7) | 50.1 (13.3) | <0.001 |
| Post | 24.0 (.) | 30.7 (9.89) | 39.0 (11.5) | 0.003 |

Supplemental table 2: Crude and adjusted risk ratios. Sex, Age, date at the start of follow-up and Charlson comorbidity index was adjusted in the model

|  | Crude Risk ratio (95% CI) | Adjusted risk ratio (95% CI) |
| --- | --- | --- |
| Pneumonia | 1.37 (1.04-1.81) | 1.23 (0.93-1.62) |
| New Stroke | 1.43 (0.96-2.14) | 1.36 (0.91-2.03) |
| New-onset heart failure | 1.67 (1.14-2.44) | 1.43 (0.97-2.10) |
| Ischemic heart disease | 1.85 (1.31-2.62) | 1.73 (1.23-2.45) |
| New-onset AF | 2.54 (1.91-3.36) | 2.45 (1.84-3.25) |
| New migraine diagnosis | 3.53 (2.49-5.00) | 3.61 (2.54-5.14) |
| Ventricular fibrillation/tachycardia | 3.75 (1.58-8.89) | 3.54 (1.48-8.43) |
| AV conduction disorder | 3.91 (2.11-7.24) | 3.60 (1.94-6.70) |
| Pacemaker implantation | 2.63 (1.57-4.40) | 2.47 (1.47-4.14) |
| Any hospitalization | 1.68 (1.53-1.86) | 1.63 (1.48-1.80) |
| CV Death | 1.25 (0.80-1.96) | 1.16 (0.74-1.82) |
| Death | 0.98 (0.74-1.32) | 0.88 (0.66-1.18) |

## Supplemental figure 1. New-onset FA after the closure.

A), Age <18


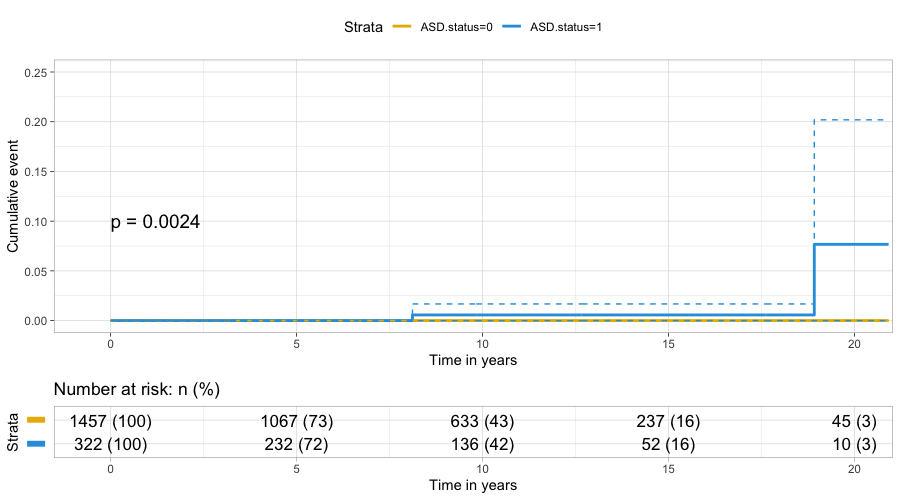


B) Age 18 to 50


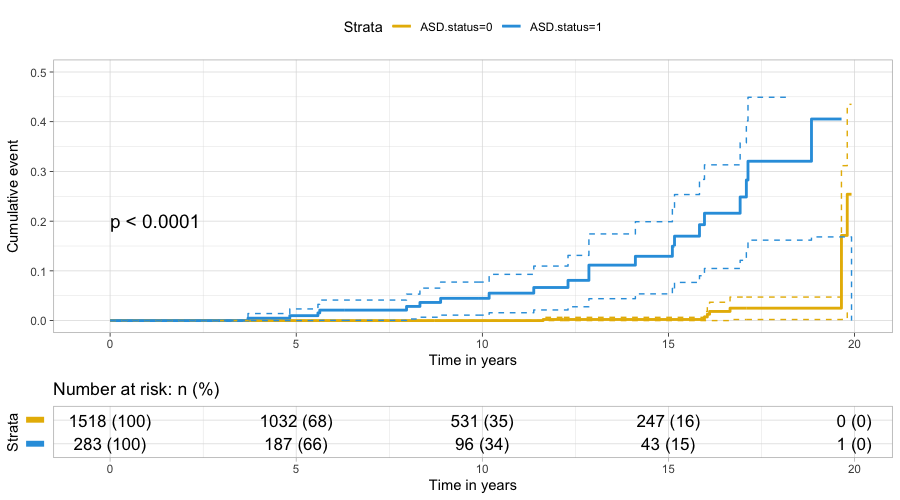


C) Age > 50


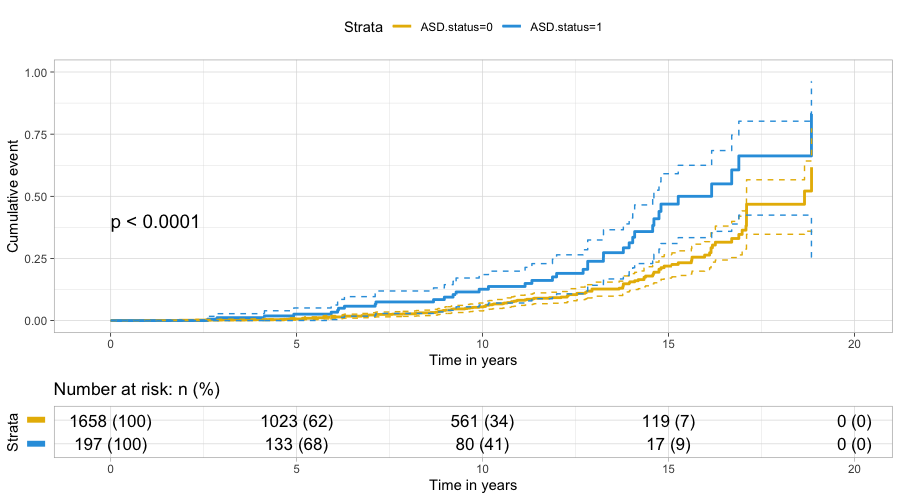


## Supplemental figure 2 New stroke after the closure

1. Age < 18


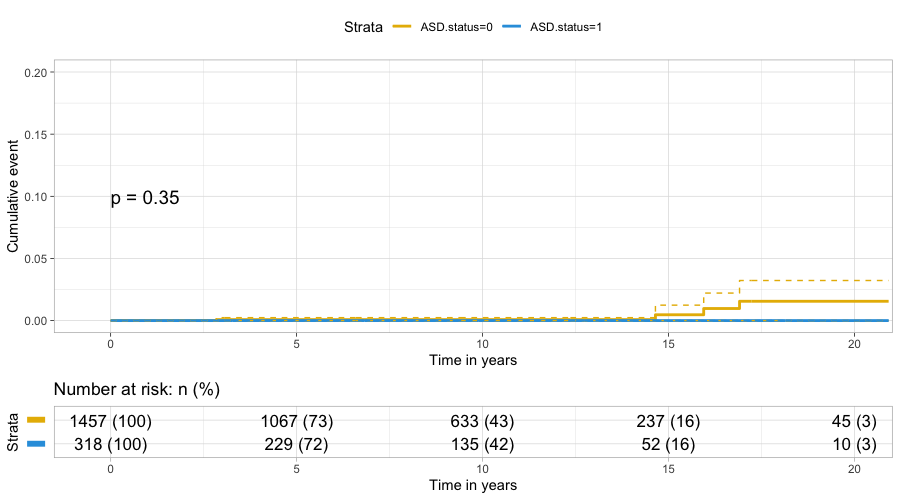


1. Age 18 to 50


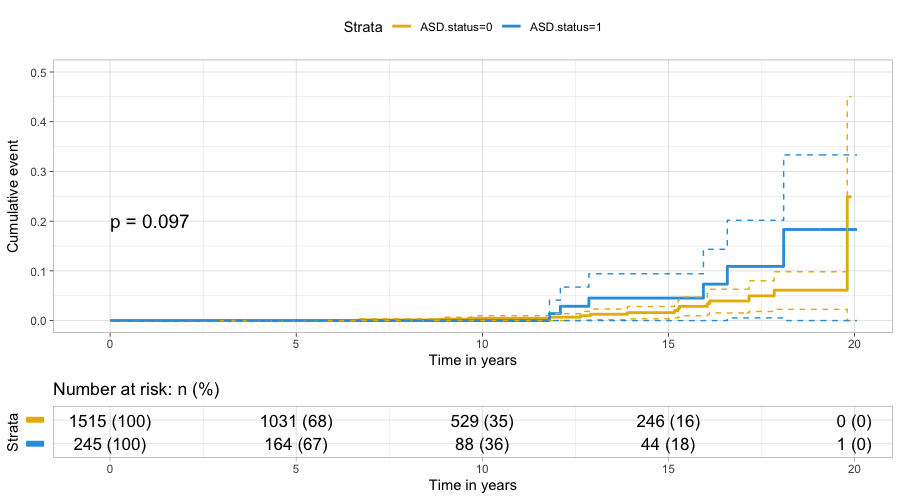


1. Age > 50


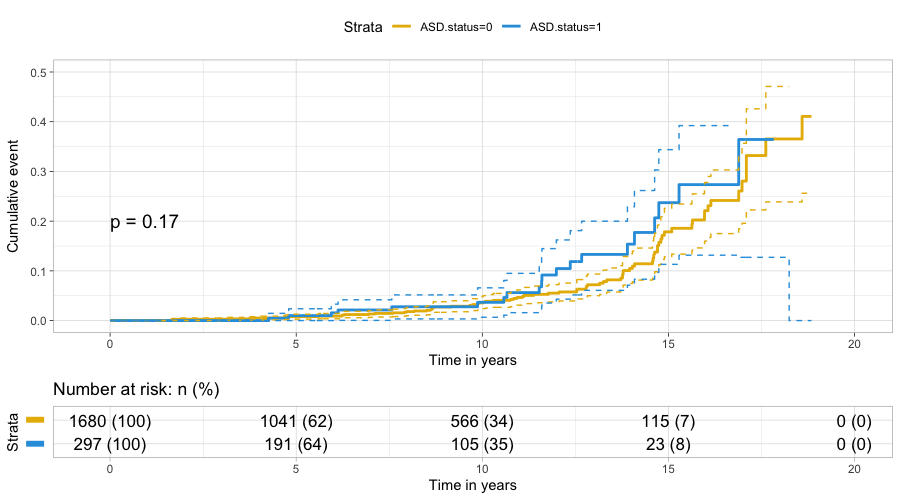


## Supplemental figure 3 New-onset migraine after the closure

1. Age < 18


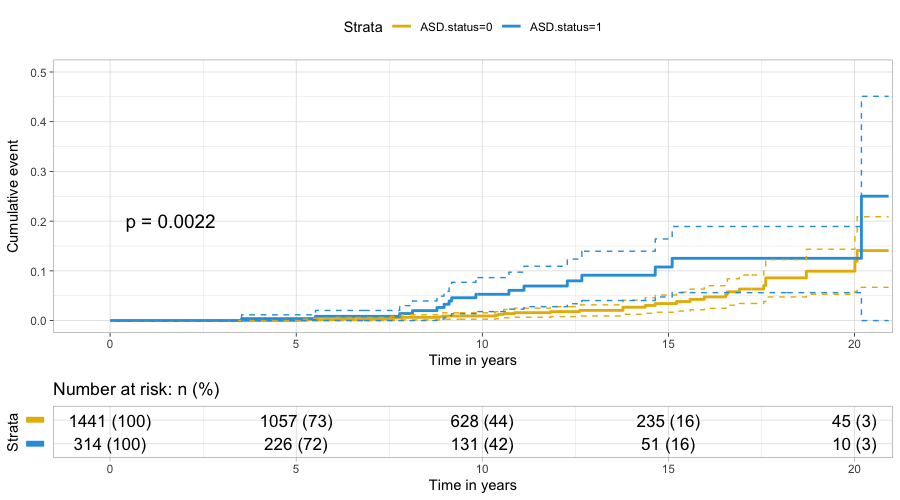


1. Age 18 to 50


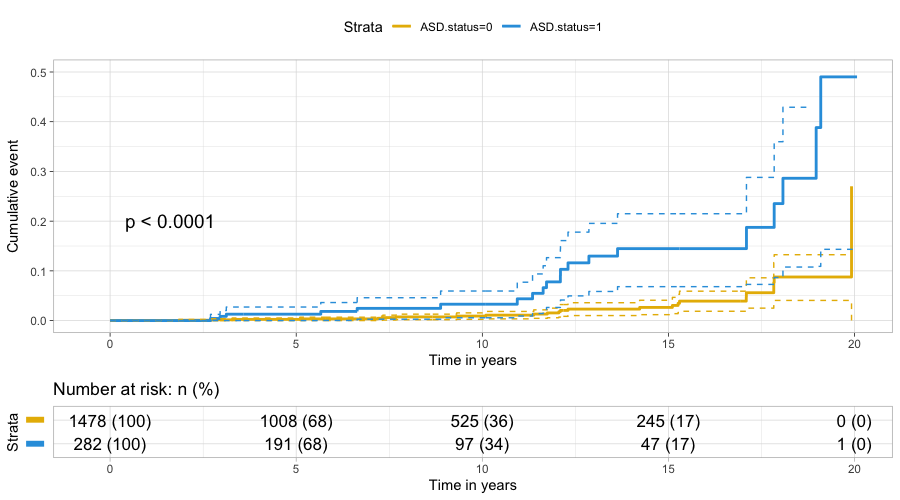


1. Age > 50


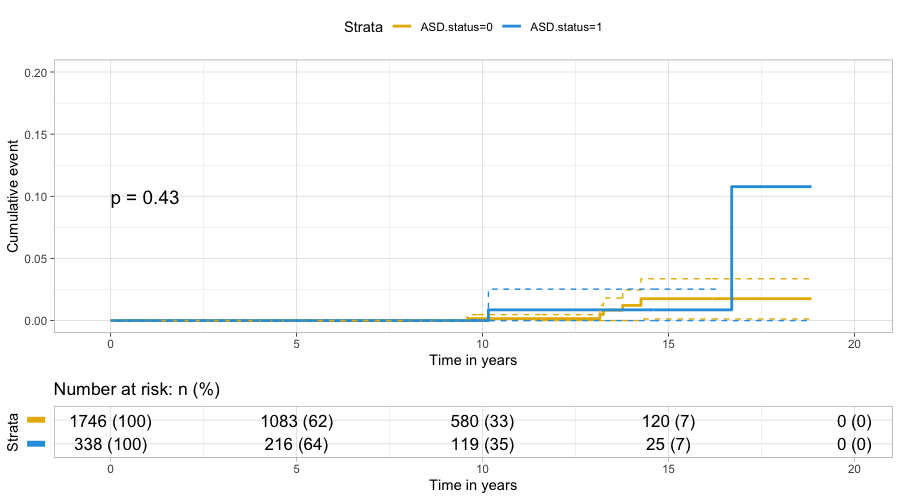


## Supplemental figure 4 New-onset heart failure after the closure

1. Age < 18


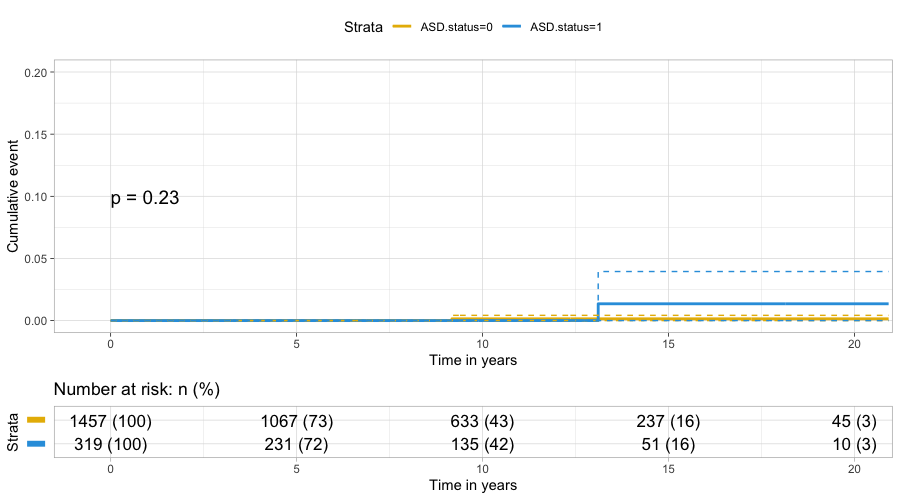


1. Age 18 to 50


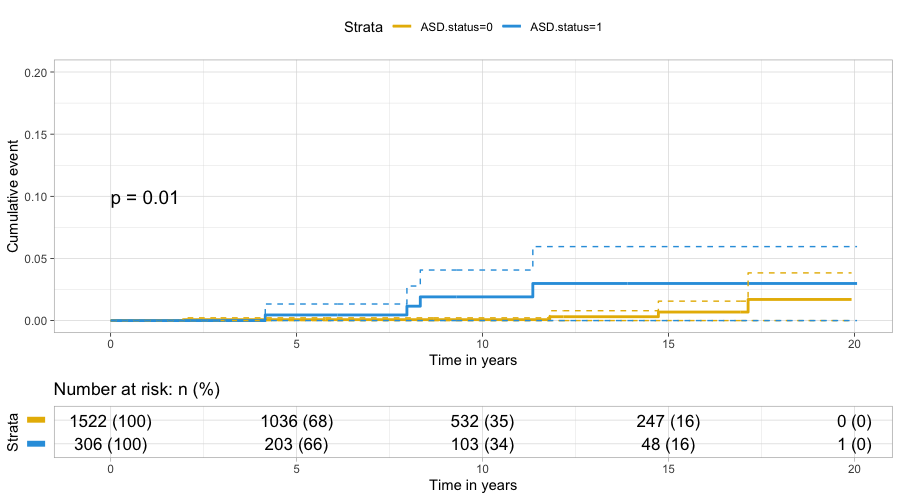


1. Age > 50


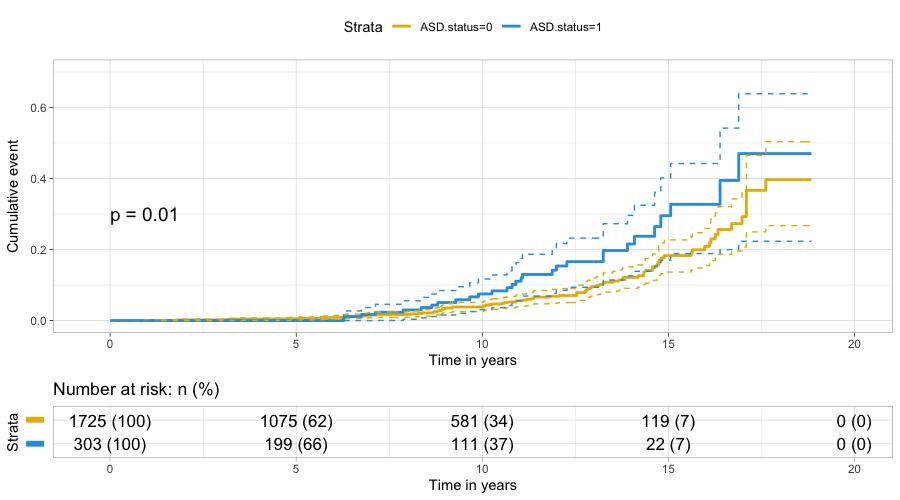

Supplement: Supplemental Material [file IANN_A_2178669_SM3252.docx]
